# Stimulatory effects of *Lycium shawii* on human melanocyte proliferation, migration, and melanogenesis: *In vitro* and *in silico* studies

**DOI:** 10.3389/fphar.2023.1169812

**Published:** 2023-04-24

**Authors:** Khalid Alghamdi, Zeyad Alehaideb, Ashok Kumar, Hamad Al-Eidi, Sahar S. Alghamdi, Rasha Suliman, Rizwan Ali, Feras Almourfi, Saleh M. Alghamdi, Mohamed Boudjelal, Sabine Matou-Nasri

**Affiliations:** ^1^ Department of Dermatology (DOD), College of Medicine (COM), King Saud University (KSU), Riyadh, Saudi Arabia; ^2^ Vitiligo Research Chair, DOD, COM, KSU, Riyadh, Saudi Arabia; ^3^ Department of Core Medical Research Facility and Platform, King Abdullah International Medical Research Center (KAIMRC), Ministry of National Guard Health Affairs (MNGHA), King Saud Bin Abdulaziz University for Health Sciences (KSAU-HS), Riyadh, Saudi Arabia; ^4^ Cell and Gene Therapy Group, Medical Genomics Research Department, KAIMRC, KSAU-HS, MNGHA, Riyadh, Saudi Arabia; ^5^ Department of Pharmaceutical Sciences, College of Pharmacy, KSAU-HS, KAIMRC, MNGHA, Riyadh, Saudi Arabia; ^6^ Department of Pharmacy, Fatima College of Health Sciences, Abu Dhabi, United Arab Emirates; ^7^ Research Centre, King Fahad Medical City, Riyadh, Saudi Arabia; ^8^ Blood and Cancer Research Department, KAIMRC, KSAU-HS, MNGHA, Riyadh, Saudi Arabia

**Keywords:** *Lycium shawii*, human melanocyte, melanogenesis, melanin, natural product, skin depigmentation, vitiligo

## Abstract

There is no first-line treatment for vitiligo, a skin disease characterized by a lack of melanin produced by the melanocytes, resulting in an urgent demand for new therapeutic drugs capable of stimulating melanocyte functions, including melanogenesis. In this study, traditional medicinal plant extracts were tested for cultured human melanocyte proliferation, migration, and melanogenesis using MTT, scratch wound-healing assays, transmission electron microscopy, immunofluorescence staining, and Western blot technology. Of the methanolic extracts, *Lycium shawii* L. (*L. shawii*) extract increased melanocyte proliferation at low concentrations and modulated melanocyte migration. At the lowest tested concentration (i.e., 7.8 μg/mL), the *L. shawii* methanolic extract promoted melanosome formation, maturation, and enhanced melanin production, which was associated with the upregulation of microphthalmia-associated transcription factor (MITF), tyrosinase, tyrosinase-related protein (TRP)-1 and TRP-2 melanogenesis-related proteins, and melanogenesis-related proteins. After the chemical analysis and *L. shawii* extract-derived metabolite identification, the *in silico* studies revealed the molecular interactions between Metabolite 5, identified as apigenin (4,5,6-trihydroxyflavone), and the copper active site of tyrosinase, predicting enhanced tyrosinase activity and subsequent melanin formation. In conclusion, *L. shawii* methanolic extract stimulates melanocyte functions, including melanin production, and its derivative Metabolite 5 enhances tyrosinase activity, suggesting further investigation of the *L. shawii* extract-derived Metabolite 5 as a potential natural drug for vitiligo treatment.

## 1 Introduction

Melanin is one of the most widely distributed families of tyrosine-based natural pigments in plants (i.e., allomelanin), microorganisms, and animals (i.e., eumelanin, pheomelanin, and neuromelanin), giving a characteristic color varying from yellow to black (Huang et al., 2018; Glagoleva et al., 2020). The colors are determined by the type, amount, and arrangement of melanin. Melanin is synthesized by specialized dendritic melanocytes, primarily located in the skin, hair bulbs, and eyes (i.e., iris and choroid coat) in humans (Maeda et al., 2015; Sitiwin et al., 2019; Moreiras et al., 2021). Melanin production (i.e., melanogenesis), occurring in subcellular lysosome-like melanosomes, is initiated by extrinsic and intrinsic factors, such as ultraviolet light exposure, steroids (i.e., androgens and alpha-melanocyte-stimulating hormone), predetermined genetic factors (i.e., age and ethnicity), and inflammatory mediators. These stimulatory factors enhance the tyrosinase activity and the rate-limiting copper-containing metalloenzyme catalyzing hydroxylation of L-tyrosine to L-DOPA, the two main substrates contributing to melanogenesis (D’Mello et al., 2016). Melanin exhibits crucial functions for skin homeostasis as a photoprotector, temperature regulator, metal chelator, and free radical scavenger, making its production a fundamental therapeutic strategy for skin diseases, including vitiligo.

Vitiligo is an autoimmune disorder of unknown etiology, leading to common depigmentation, characterized by white patches caused by melanocyte loss, which affects about 1% of the global population (Abdel-Malek et al., 2020). The highest prevalence is in European, American, South Asian, and Japanese populations (Said-Fernandez et al., 2021; Bibeau et al., 2022). These white patches can disturb the cosmetic aspect and psychosocial functioning, which could result in depression (Cupertino et al., 2017; Varma et al., 2019). Vitiligo is treated by several modalities, including phototherapy (i.e., psoralen and ultraviolet A (PUVA); 8-methoxypsoralen; narrowband ultraviolet B (NB-UVB); laser therapy (i.e., 308-nm xenon chloride excimer laser); surgical therapy (i.e., melanocyte-rich tissue grafting and melanocyte grafting); and immunosuppression therapy (i.e., corticosteroids) (Frisoli and Harris, 2017; Kawakami, 2022). Multiple surgical modalities have offered patients significant benefits, but the effectiveness of these treatments, especially in the long term, remains distressingly poor, and the side effects are a cause for concern (Abdel-Malek et al., 2020; Lotti et al., 2020). Transplantation of autologous cultured melanocytes was reported to successfully re-pigment vitiliginous skin lesions in a variety of settings (Zokaei et al., 2019). This procedure requires an adequate number of melanocytes to efficiently re-pigment the depigmented macules. Unfortunately, routine culture methods require substantial time to provide a sufficient number of melanocytes for autologous transplantation (Silpa-Archa et al., 2017; Zhang et al., 2021). An appropriate treatment method is urgently needed.

Despite major scientific advances in chemistry, plant-derived medicinal drugs significantly contribute to drug discovery and continue to be an important source for combating serious diseases (Khan et al., 2019; Ogunrinola et al., 2021). In Saudi Arabia, there is a rich tradition of the use of herbal medicine for the treatment of various diseases, such as inflammation, infection, cancer, and skin diseases, including vitiligo (Al-Harbi, 2017; Shahat et al., 2017; Al-snafi, 2018; Alzandi et al., 2021). Despite some investigations in the last decade, less number of traditionally used medicinal plants have been evaluated for their effect against skin disorders such as vitiligo, as well as for their chemical properties (Al-Harbi, 2017; Shahat et al., 2017; Narayanaswamy and Ismail, 2018). A few herbs and constituents, including glycyrrhizin (Lee et al., 2005), kava (*Piper methysticum* G. Forst) rhizome extract (Matsuda et al., 2006), quercetin (Mitsunaga and Yamauchi, 2015), and fraxinol (Moon et al., 2022), increase melanogenesis in B16 melanoma cells, indicating that the natural resources should be extensively screened for the development of gray hair-preventing agents or therapeutic drugs to induce re-pigmentation in the skin of vitiligo patients. In addition, stimulants of melanocyte proliferation and migration are potential treatments for vitiligo as well (Li et al., 2020). We hypothesize that several medicinal plants, if used at a specific concentration, may enhance the proliferation rate, migration, and melanogenesis of melanocytes *in vitro*.

## 2 Materials and methods

### 2.1 Chemicals and reagents

Medium 254, human melanocyte growth supplement (HMGS); Dulbecco’s modified Eagle medium (DMEM); fetal bovine serum (FBS); antibiotic–antimycotic (i.e., 100 μg/mL streptomycin, 100 IU/mL penicillin, and 25 μg/mL amphotericin B); trypsin 0.25%/ethylenediaminetetraacetic acid (EDTA); and Dulbecco’s phosphate-buffered saline (PBS) were provided from Gibco^®^ (Waltham, MA). Nonylphenoxypolyethoxyethanol (NP40) cell lysis buffer was purchased from Invitrogen (Waltham, MA). Methanol (chromatography grade, ≥99%) was obtained from Honeywell Riedel-de Haen (Seelze, Germany). Purified carbon dioxide (CO_2_) gas was provided by Saudi Industrial Gas (Dammam, Saudi Arabia). Ultrapure water was produced using a Millipore (Billerica, MA) system with a resistivity reading of 18.2 MΩ cm at 25°C. Primary mouse monoclonal antibodies directed against tyrosinase-related protein (TRP)1 conjugated to fluorescein isothiocyanate (FITC) (clone TA99, cat. #sc-58438); TRP-2/DOPAchrome tautomerase conjugated to phycoerythrin (PE) (DCT, clone E-10, cat. #sc-166716); tyrosinase (clone T311, cat. #sc-20035); TRP-1 (clone G-9, cat. #sc-166857); TRP-2 (clone C-9, cat. #sc-74439); and microphthalmia-associated transcription factor (MITF) (clone 21D1418, cat. #sc-52938) were provided by Santa Cruz Biotechnology (Dallas, TX). All other reagents, unless otherwise mentioned, were purchased from Sigma-Aldrich (St Louis, MO).

### 2.2 Collection of plant-based natural products

*Anastatica hierochuntica* L. (*A. hierochuntica*) (aerial part, *Brassicaceae* family); *Calligonum comosum* L'Hér. (*C. comosom*) (aerial part, *Polygonaceae* family); *Lycium shawii* L. (*L. shawii*) (leaves, Solanaceae family), and *Rhazya stricta* Decne (*R. stricta*) (leaves, *Apocynaceae* family) were purchased from local shops in Riyadh, Saudi Arabia. *Achillea fragrantissima* (Forssk.) Sch.Bip. (*A. fragrantissima*) (aerial part, *Asteraceae* family) and *Calotropis procera* (Aiton) Dryand. (*C. procera*) (leaves, *Apocynaceae* family) were wild-crafted from the desert oasis of Rhodat Khoraim, near Ramah city, in the central region of Saudi Arabia (coordinates: 25°23′16.1″N 47°16′08.6″E). The collected plants (listed in Table 1) were authenticated by Professor Mona Alwhibi, Department of Botany and Microbiology, King Saud University, Riyadh, Saudi Arabia. The fresh aerial parts or leaves were rinsed with filtered water and left to dry under a stream of warm dry air. The dried natural products were finely powdered using an electric motor grinder and kept in dark at room temperature until extraction.

**TABLE 1:** List of the medicinal plants.

Plant family	Scientific name	Common name (Arabic)	Plant part	Herbium accession number
*Asteraceae*	*Achillea fragrantissima* (Forssk.) Sch.Bip. (*A. fragrantissima*)	Qaisoom ( 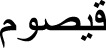 )	Arial	23389
*Brassicaceae*	*Anastatica hierochuntica* L. (*A. hierochuntica*)	Kaff Maryam ( 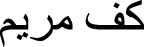 )	Arial	24327
*Polygonaceae*	*Calligonum comosum* L’Hér. (*C. comosom*)	Arta ( 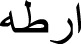 )	Arial	24328
*Apocynaceae*	*Calotropis procera* (Aiton) Dryand. (*C. procera*)	Eshar ( 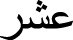 )	Leaves	24319
*Solanaceae*	*Lycium shawii* L. (*L. shawii*)	Awsaj ( 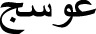 )	Leaves	24085
*Apocynaceae*	*Rhazya stricta* Decne (*R. stricta*)	Harmal ( 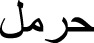 )	Leaves	24321

### 2.3 Methanolic extraction of plant-based natural products

Methanol was utilized as a solvent for the rapid extraction of low-molecular constituents from plant-based natural products, as commonly used (Wagner et al., 2011). Approximately 500.0 mg of dried natural product powder was extracted using 10.0 mL of methanol under high-power sonication using a Sonics (Newton, CT, United States) Vibra-Cell™ ultrasonic liquid processor (model GEX-130 probe-sonicator) for 30 min. The sonicated extracts were filtered using a Sartorius Stedim Biotech (Göttingen, Germany) quantitative ashless paper filter under gravity flow and dried in an incubator at 40°C. The remaining dried pellets were weighted and reconstituted with 0.5 mL of dimethyl sulfoxide (DMSO) by vortexing until completely dissolved. The reconstituted extracts were stored at a cool temperature in the dark until use.

### 2.4 Human melanocyte isolation and expansion

The methods used for the collection and culture of the primary human melanocytes from fresh brown foreskins excised during infant circumcision were similar to those of our previous publications, with minor modifications (AlGhamdi et al., 2015; AlGhamdi et al., 2020). Briefly, the neonatal foreskin tissues were kept in a transport medium (DMEM, 10% FBS, and antibiotics/antimycotic) and transferred to a melanocyte culture laboratory. The tissues were washed thrice with sterile PBS; cut into small pieces (5 × 5 mm); and then placed in PBS containing 0.25% trypsin/EDTA solution, followed by incubation at 37°C for 1 h. The epidermal sheets were separated from the dermis, and the pieces were scraped with a sterile scalpel blade to obtain a maximum number of melanocytes from the basal layer of the epidermis. The cells were harvested by centrifugation (200 ×*g* for 3 min at room temperature) and seeded in a tissue culture flask with melanocyte growth-promoting medium, composed of medium M254, supplemented with HMGS. After 7–10 days, the semi-confluent monolayer culture of melanocytes was trypsinized, as aforementioned, to collect the melanocytes and was re-seeded for cell expansion. The third and fourth passage melanocytes were used throughout the study.

### 2.5 Primary human melanocyte treatment

The primary human melanocytes were seeded (1.5 × 10^4^/cm^2^) in sterile plates and incubated overnight. The next day, the medium was replaced with various concentrations of the plant extract diluted in a melanocyte growth-promoting medium. A stock of plant extracts was prepared in DMSO, and the melanocyte growth-promoting medium was used to prepare serial solutions for the final concentrations of the plant extracts as follows: 7.8, 15.6, 31.3, 62.5, 125.0, 250.0, and 500.0 μg/mL. The melanocytes exposed to the melanocyte growth-promoting medium without any plant extracts were the control (i.e., untreated melanocytes). The plant extract-treated cells and untreated cells were processed for downstream applications. 1% DMSO-treated cells were used as a negative control throughout the study, serving as a solvent for the reconstitution of the dried methanolic extract.

### 2.6 Fluorescence microscopy for TRP-1 and TRP-2 cellular localization

The melanocytes were seeded in an 8-well chambered µ-slide (ibidi GmbH, Gräfelfing, Germany) overnight at 37°C and 5% CO_2_. The following day, the cells were washed and fixed with 4% paraformaldehyde for 10 min. The fixed cells were incubated with anti-TRP-1-FITC and anti-TRP-2/DCT-PE antibodies overnight. Finally, the cells were counterstained with Hoechst 33342 nuclear dye solution (cat. #H3570, Invitrogen, Thermo Fisher Scientific Corp.) for 10 min. The samples were visualized with a laser scanning microscope, LSM 780 (Carl Zeiss, Germany), using the wide-field option.

### 2.7 MTT cell proliferation assay

The biological effect of methanolic extracts on melanocyte proliferation was assessed using a 3-[4,5-dimethylthiazol-2-yl]-2,5-diphenyltetrazolium bromide (MTT) assay-based cell growth determination kit (Sigma, #CGD-1), as detailed in AlGhamdi et al., (2015). Briefly, the treated and untreated melanocytes, previously seeded in 96-well plates and then incubated for 72 hrs, were exposed to a 10% MTT solution for 3 h at 37°C. The MTT solution was replaced with an equal volume of isopropanol. The plates were shaken for 45 min at room temperature. The absorbance was measured at 549 nm using a Universal Microplate Reader ELx800 counter (BioTek Instruments Inc., Winooski, VT).

### 2.8 Melanocyte migration assay

The effect of the different doses of *L. shawii* on melanocyte migration was assessed with the *in vitro* scratch wound-healing assay (Liang et al., 2007), with slight modifications, as mentioned in AlGhamdi et al. (2020). The melanocytes were seeded and incubated overnight. After which, a scratch was created in a cell monolayer using a sterile 200-µL pipette tip. The cell debris was removed by changing the media, and the melanocytes were exposed to different doses of *L. shawii* extract. Images were captured at 72 h during the migration to cover the scratch wound area. Photomicrographs were taken using a charge-coupled device (CCD) camera attached to an inverted stage-contrast microscope (Olympus, Tokyo, Japan) at a magnification of 10x.

### 2.9 Transmission electron microscopy for melanosome formation and maturation

The melanocyte samples were prepared for transmission electron microscopy (TEM) according to the published protocol in Ali et al. (2021). Briefly, the cells were fixed in 4% glutaraldehyde and dehydrated using graded concentrations of ethanol. The samples were then embedded in an embedding medium, and ultra-thin sections were produced using a Boeckeler Instruments ultramicrotome (Tucson, AZ, United States). The sections were mounted on copper grids and stained for contrast with 1% uranyl acetate and lead citrate. The TEM images were acquired using the JEM-1400 (JEOL Ltd., Akishima, Japan). The acquired melanocyte images of samples of five patients at different magnifications, i.e., 8,000x and 40000x, were randomly selected to quantify the melanosomes using ImageJ software v1.46r. The ImageJ multipoint tool was applied for labeling and counting the forming melanosomes. The number of melanosomes undergoing the four stages of maturation was counted based on different colors and structure codes for each stage. In each sample, ten random melanocytes were chosen for melanosome counting and categorization.

### 2.10 Melanin quantification

The melanin content was measured according to a method established by Fernandes et al. (2016), with modifications. Briefly, after 24 h of incubation post-treatment, the untreated and treated cells were trypsinized and centrifuged at 1,000×*g* for 10 min. The cell pellets were washed twice with PBS. The supernatants were discarded, and the cells were suspended in 0.5 mL of 1 M NaOH containing 10% DMSO and stored at −80°C until analysis.

Before the analysis, the suspensions were heated at 80°C for 1 h with periodic vortexing, after which the suspensions were centrifuged at 3,000×*g* for 5 min. Exactly 0.3 mL of the supernatant was added in an Eppendorf tube for melanin measurement. H_2_O_2_ (50%, *w/v*) was added at a final concentration of 30% (*v/v*) and left for incubation in a dark room at room temperature for 4 h. After centrifugation at 3,000×*g* for 5 min, the samples were placed in a 96-well plate in equal volumes and measured using a Molecular Devices SpectraMax M5 fluorometer (San Jose, CA, United States), set at 470 nm excitation and 550 nm emission. The melanin concentration was extrapolated from a generated melanin standard curve.

### 2.11 Protein extraction and Western blotting analysis

The extraction, separation, and detection of melanogenesis-related proteins, including MITF, tyrosinase, TRP-1, and TRP-2, were performed according to our previous method (Alehaideb et al., 2021), with modifications. Briefly, after 72 h of incubation, the untreated and treated cells were trypsinized, collected, and rinsed twice with PBS. The cell pellets were extracted once with NP40 buffer, and the protein concentrations were determined using a Qubit^®^ protein assay kit (Invitrogen). Cell lysates (150.0 μg) were loaded on 7% sodium dodecyl sulfate-polyacrylamide gel electrophoresis. The separated proteins were detected and quantified using primary mouse monoclonal antibodies directed against melanogenesis-related proteins including tyrosinase, TRP-1, TRP-2, and MITF. Mouse monoclonal (clone 236–10501, #A11126, Thermo Fisher Scientific) and rabbit polyclonal (#ab18251, Abcam, Cambridge, MA) anti-α tubulin antibodies were loading controls. Infrared fluorescent dye IRDye^®^ 800CW-conjugated goat anti-mouse antibody (#926–32210) and IRDye^®^ 680RD-conjugated goat anti-rabbit antibody (#926–68071) were used as secondary antibodies (LI-COR Biosciences, Lincoln, NE). The blots containing the targeted proteins were scanned on the LI-COR Odyssey CLx, and the protein expression levels were analyzed using ImageJ software.

### 2.12 Metabolite identification using LC-QTOF

*L. shawii* metabolites were separated and tentatively identified using an Agilent (Santa Clara, CA) 1,260 Infinity high-performance liquid chromatography (LC) system coupled to an Agilent 6,530 quadrupole time-of-flight (QTOF), as described in Alehaideb et al. (2022). Briefly, the separation was performed on an Agilent SB-C18 column (4.6 mm × 150 mm, 1.8 μm particle size) with the gradient elution: 0–2 min, 5% B; 2–17 min, 5%–100% B; 17–21 min, 95% B; 21–25 min, 5% B, using mobile-phase A (0.1% formic acid in water) and mobile-phase B (0.1% formic acid in methanol). The injection volume was 10 μL, and the flow rate was 250 μL/min. The gas temperature was set at 300°C, the gas flow at 8 L/min, the nebulizer pressure at 35 psi, the sheath gas flow rate at 11 L/min, the gas temperature at 350°C, and the scanning range was set from 50 to 800 (*m/z*). The data were generated by the Agilent MassHunter (version B.06.00) analysis software.

### 2.13 Computational docking study

To predict the likely molecular interactions between the identified metabolites and the enzyme tyrosinase, Maestro Schrödinger software was applied. For each metabolite, the SMILES (simplified molecular-input line-entry system) was used as an input to generate the two-dimensional (2D) structure, followed by conversion into the three-dimensional (3D) structure with minimization, using the LigPrep tool. The crystal structure of tyrosinase of *Agaricus bisporus* was downloaded from the Protein Data Bank (PDB) website with PDB ID: 2Y9X (resolution: 2.78 Å). The enzyme was prepared, optimized, and minimized using the Protein Preparation Wizard tool, and the grid for the active site containing tropolone was generated. The metabolites were docked into the generated grid using the Glide tool, and each docking pose was subjected to a post-docking analysis (Maestro Schrödinger, Release 2022-2).

### 2.14 Predictions of absorption, distribution, metabolism, and excretion (ADME) properties

Multiple pharmacokinetic parameters and cytochrome P450 (CYP) enzyme inhibition were predicted with the SwissADME web server (http://www.swissadme.ch/) and the QikProp tool in Maestro. The pharmacokinetic features, important for drug discovery, were selected, including molecular weight, lipophilicity (Log Po/w), solubility (Log S), blood–brain barrier (BBB) penetration, and human oral absorption. The identified metabolites were also evaluated for their ability to inhibit several CYP enzymes such as CYP1A2, CYP2C19, CYP2C9, CYP2D6, and CYP3A4 that are crucial for drug metabolism (Daina et al., 2017).

### 2.15 Organ toxicity and safety predictions

The identified metabolites were evaluated and assessed for their toxicity profile using the ProTox-II webserver (https://tox-new.charite.de/protox_II/). The toxicity of each metabolite was evaluated and computed for several endpoint toxicity predictions, including hepatotoxicity, cytotoxicity, carcinogenicity, mutagenicity, and immunotoxicity (Banerjee et al., 2018).

### 2.16 Statistical analysis

The experimental data are presented as mean ± standard deviation (SD) of three independent assays. Student's t-test or one-way ANOVA, followed by Tukey’s *post hoc* test, was applied to determine the statistical differences between the experimental conditions. The values of *p* ≤ 0.05 (*) or *p* ≤ 0.01 (**) were considered statistically significant.

## 3 Results

### 3.1 Screening of plant-derived methanolic extracts with stimulatory effects on melanocyte proliferation and migration

**FIGURE 1:**
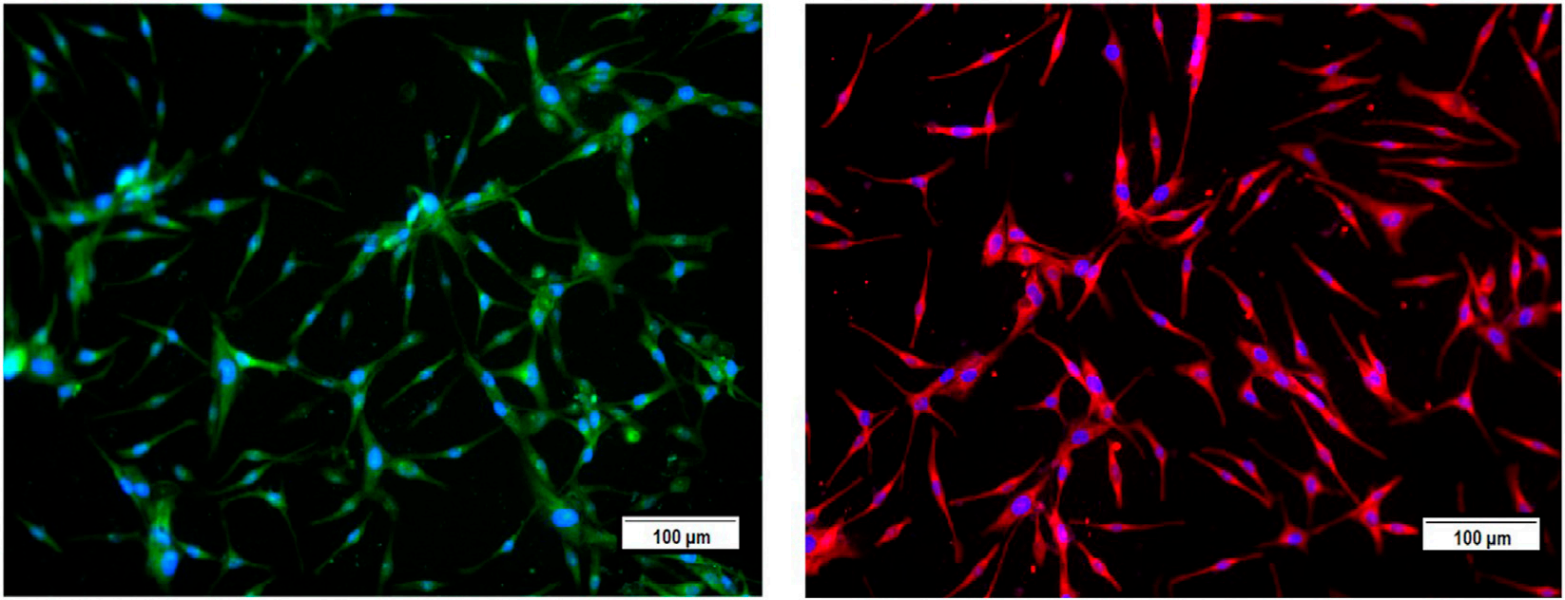
Characterization of the primary culture of melanocytes from neonatal foreskin by detecting the expression of melanosomal glycoproteins TRP-1 (left) and TRP-2 (right) using immunofluorescence staining.

In this study, we screened several traditional herbal medicines from the Middle Eastern region for their potential stimulatory effects on neonatal foreskin-derived melanocyte proliferation, migration, and melanogenesis. Isolated and expanded from the region of the neonatal foreskin, the primary culture of the human melanocytes was characterized by their typical cell morphology, giving a spindle-shaped dendritic appearance, and by the expression of the main melanocyte protein markers, including TRP-1 and TRP-2. Representative photomicrographs confirmed the abundant expression of both intracellular melanosomal glycoproteins TRP-1 and TRP-2, with widespread cytoplasmic localization for TRP-1 and more perinuclear localization for TRP-2 (Figure 1).

**FIGURE 2:**
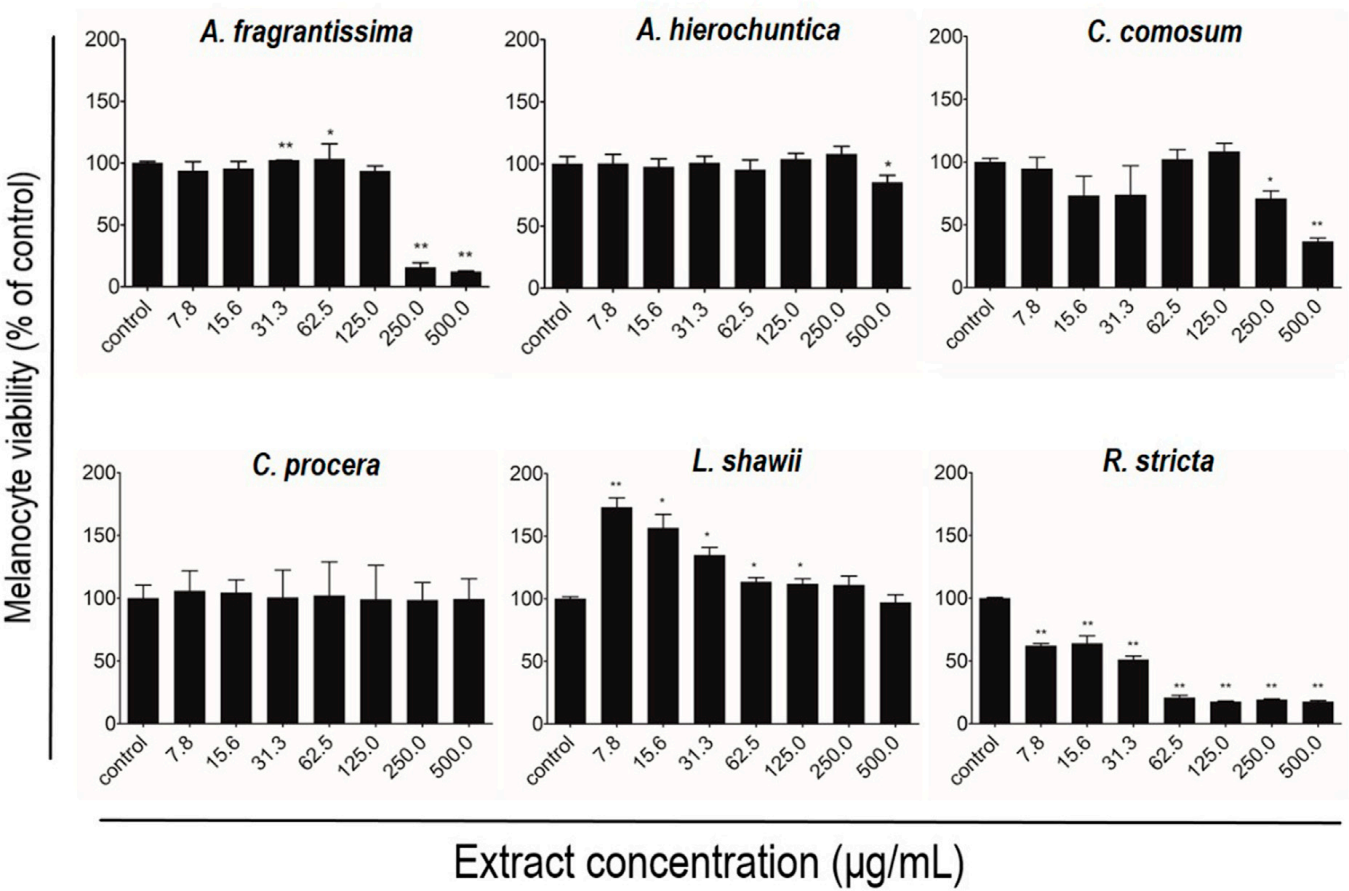
Modulatory effects of screened plant extracts on the proliferation of primary human melanocytes using MTT assay. Melanocytes were treated with various concentrations 7.8–500 μg/mL) of different methanolic extracts for 72 h of incubation. Cell viability was assessed using the MTT assay and expressed as a percentage of control, the untreated melanocyte viability, corresponding to 100%. The results are presented as mean ± SD of three independent experiments. *p ≤ 0.05 and **p ≤ 0.01 signify a statistically significant difference compared with the control.

**FIGURE 3:**
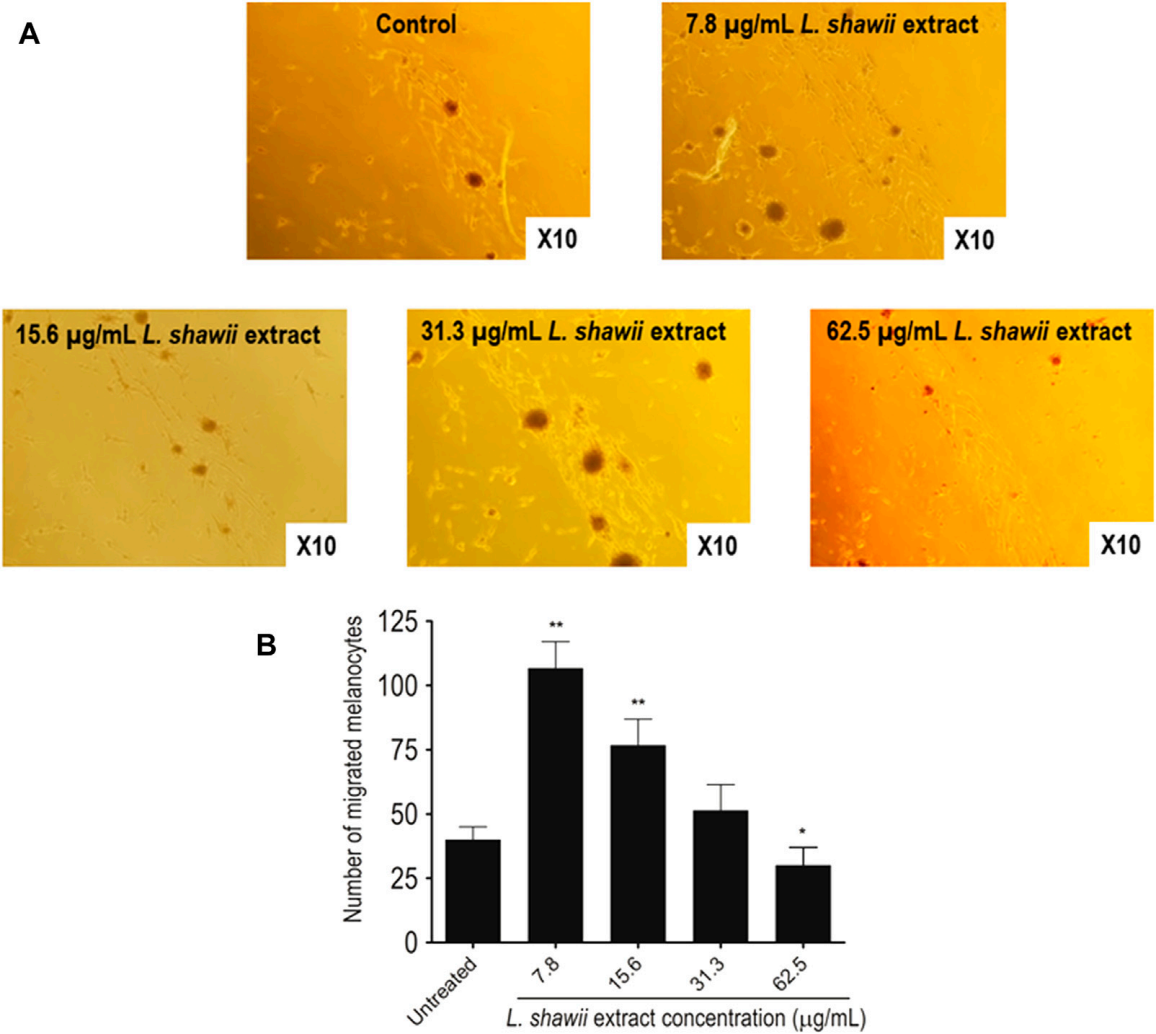
Modulatory effects of various concentrations of *L. shawii* extract on primary human melanocyte migration using the scratch wound-healing assay. **(A)** Representative photomicrographs showing melanocyte migration covering the scratch wound area after 72 h of incubation in the presence of various concentrations (7.8–31.3 μg/mL) of *L. shawii* extract, compared with the control, the untreated cells. **(B)** The bar graph indicates the number of migrated melanocyte and the results are presented as mean ± SD of three independent experiments. *p ≤ 0.05 and **p ≤ 0.01 signify a statistically significant difference compared with the control.

After melanocyte isolation and characterization, a series of experiments were conducted for evaluating the biological effects of the selected plant extracts, tested at various concentrations regarding melanocyte proliferation. The *A. fragrantissima* extract showed a gradual significant increase in melanocyte proliferation by 15% at 62.5 μg/mL, compared to the untreated cells (Figure 2). The *A. hierochuntica* extract maintained an increase in melanocyte proliferation in most of the extract concentrations (7.81–250 μg/mL), reaching 15%–20%, compared to the untreated cells (Figure 2). The *C. comosum* extract showed a slight increase at 62.5 and 125.0 μg/mL, reaching 15%–20%, compared to the untreated cells (Figure 2). The *C. procera* extract exhibited no mitogenic effects at all concentrations tested. The extract of *L. shawii* increased melanocyte proliferation significantly at the lowest treatment concentration of 7.81 μg/mL, reaching 75%, and *R. stricta* showed no proliferative effects at any concentration (Figure 2). The traditional medicinal compounds of *A. fragrantissima* and *C. comosum* showed cytotoxic effects at concentrations above 125 μg/mL, and *R. stricta* exerted cytotoxic effects at concentrations above 7.8 μg/mL (Figure 2). The remaining traditional medicines had no or little cytotoxic effect up to treatment concentrations of 500 μg/mL (Figure 2). Due to its pro-proliferative effect and low cytotoxicity, the *L. shawii* extract was chosen for further investigations, including the melanocyte migration assay. Using the scratch wound-healing assay, the *L. shawii* methanolic extract showed a significant and favorable pro-migratory effect on melanocytes at low concentrations, especially at 7.8, 15.6, and 31.3 μg/mL concentrations (Figure 3). At the highest concentration tested, the *L. shawii* methanolic extract significantly inhibited melanocyte migration (Figure 3).

### 3.2 *L. shawii* extract exhibits stimulatory effects on melanosome formation and maturation

**FIGURE 4:**
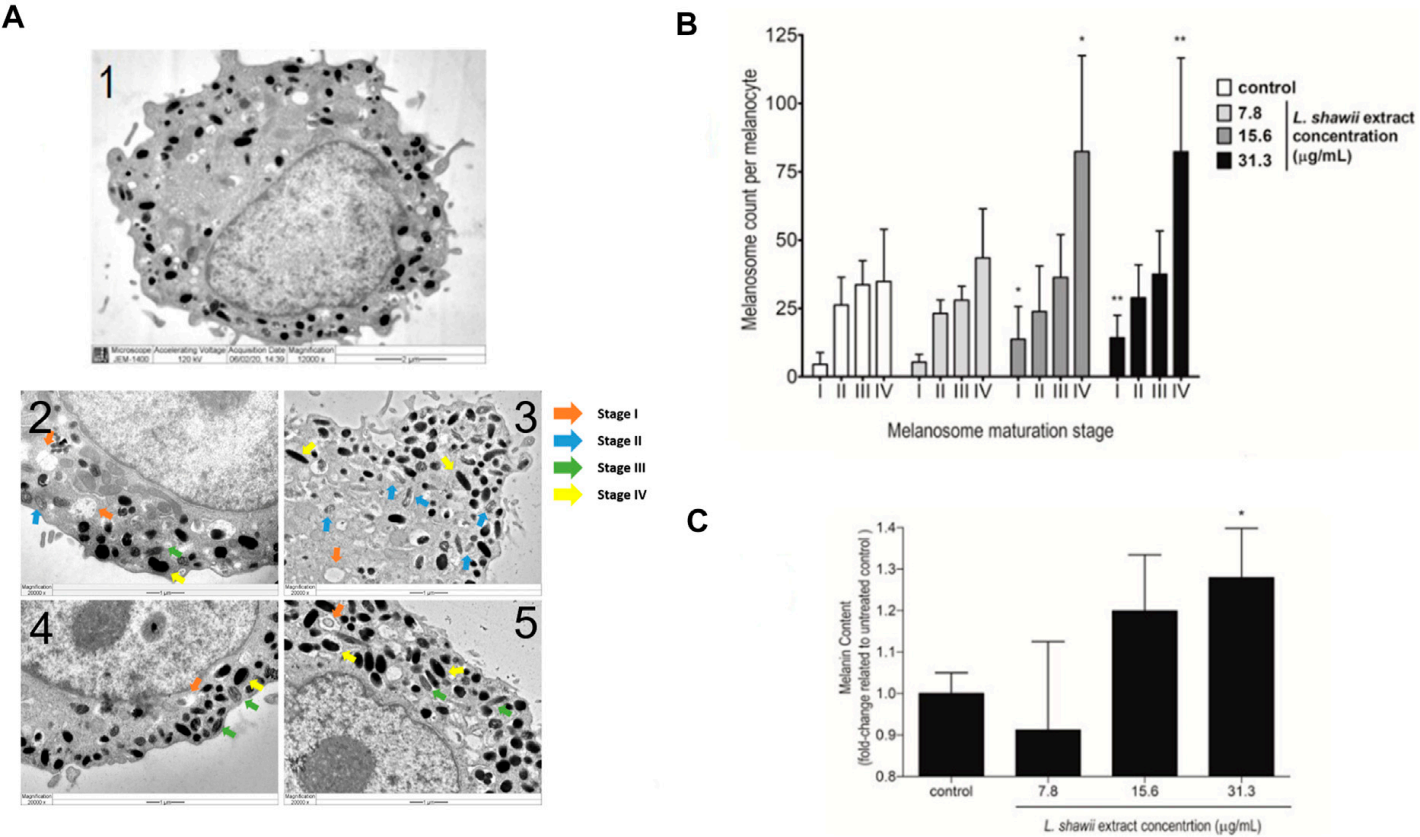
Stimulatory effects of the *L. shawii* extract on melanosome maturation and melanin production in primary human melanocytes. **(A)** Representative electron microscopic photomicrographs of a mature (1) human melanocyte showing various (I–IV) stages of melanosomes in the absence (2) or presence of 7.8 μg/mL (3), 15.6 μg/mL (4), and 31.3 μg/mL (5) of the *L. shawii* extract. The arrows indicate examples of melanosome stages as follows: stage I showing a planar clathrin coat, intraluminal vesicles, and membranous tubules; stage II characterized by intraluminal amyloid sheets; stage III showing electron-dense melanin deposits; stage IV characterized by the filling of the melanosome lumen with electron-dense melanin (Benito-Martínez et al., 2020). **(B)** The bar graph indicates the effects of the *L. shawii* extract on melanosome formation at different stages of maturation per ten separate melanocytes per each *L. shawii*-treated and untreated cells. **(C)** The bar graph shows the measurement of melanin content after treatment with the *L. shawii* methanolic extract. The results are presented as the mean ± SD of three independent experiments. *p < 0.05 and **p < 0.01 signify a statistically significant difference compared with the control.

Regarding the biological impact of the *L. shawii* extract on melanogenesis of the primary human melanocytes, TEM analysis was performed after 72 h of cell exposure to various concentrations (i.e., 7.8, 15.6, and 31.3 μg/mL) of the *L. shawii* extract. The melanogenesis was assessed by determining the number of melanosomes counted at different stages (i.e., I, II, III, and IV) of maturation. According to the literature (Moreiras et al., 2021; Tian et al., 2021), at stages I and II, the immature and non-pigmented melanosomes were observed to contain endosomal compartments called coated endosomes and internal striations, respectively (Figure 4A). Stage III was characterized by melanin pigment deposition onto the striations, and stage IV was fully melanized (Figure 4A). The untreated melanocytes contained the least number of stage I melanosomes, and higher numbers of melanosomes were counted, which increased with the stages of maturation (Figure 4B). At the lowest tested concentration of the *L. shawii* extract, no significant difference was observed between the number of melanosomes at stage I, stage II, and stage III (Figure 4B). However, the number of stage IV melanosomes was increased in the treated melanocytes compared to the number of stage IV melanosomes counted in the untreated melanocytes (Figure 4B). At the highest concentrations (i.e., 15.6 and 31.3 μg/mL) of the *L. shawii* extract, the number of stage I melanosomes tripled compared to the number of stage I melanosomes counted in the untreated and 7.8 μg/mL *L. shawii* extract-treated melanocytes (Figure 4B). Compared to the untreated melanocytes and the melanocytes exposed to the lowest concentration of *L. shawii* extract, no change in the number of stage II and stage III melanosomes was observed, but the number of stage IV melanosomes doubled (Figure 4B). Using a fluorometer, the melanin content produced by each melanocyte displayed a significant increase in melanin concentration that was only noticed at the highest concentration (i.e., 31.3 μg/mL) of the *L. shawii* extract, compared with the untreated cells (Figure 4C).

### 3.3 Stimulation of the expression of melanogenesis-related proteins MITF, tyrosinase, TRP-1, and TRP-2 and melanin concentration by the *L. shawii* extract

**FIGURE 5:**
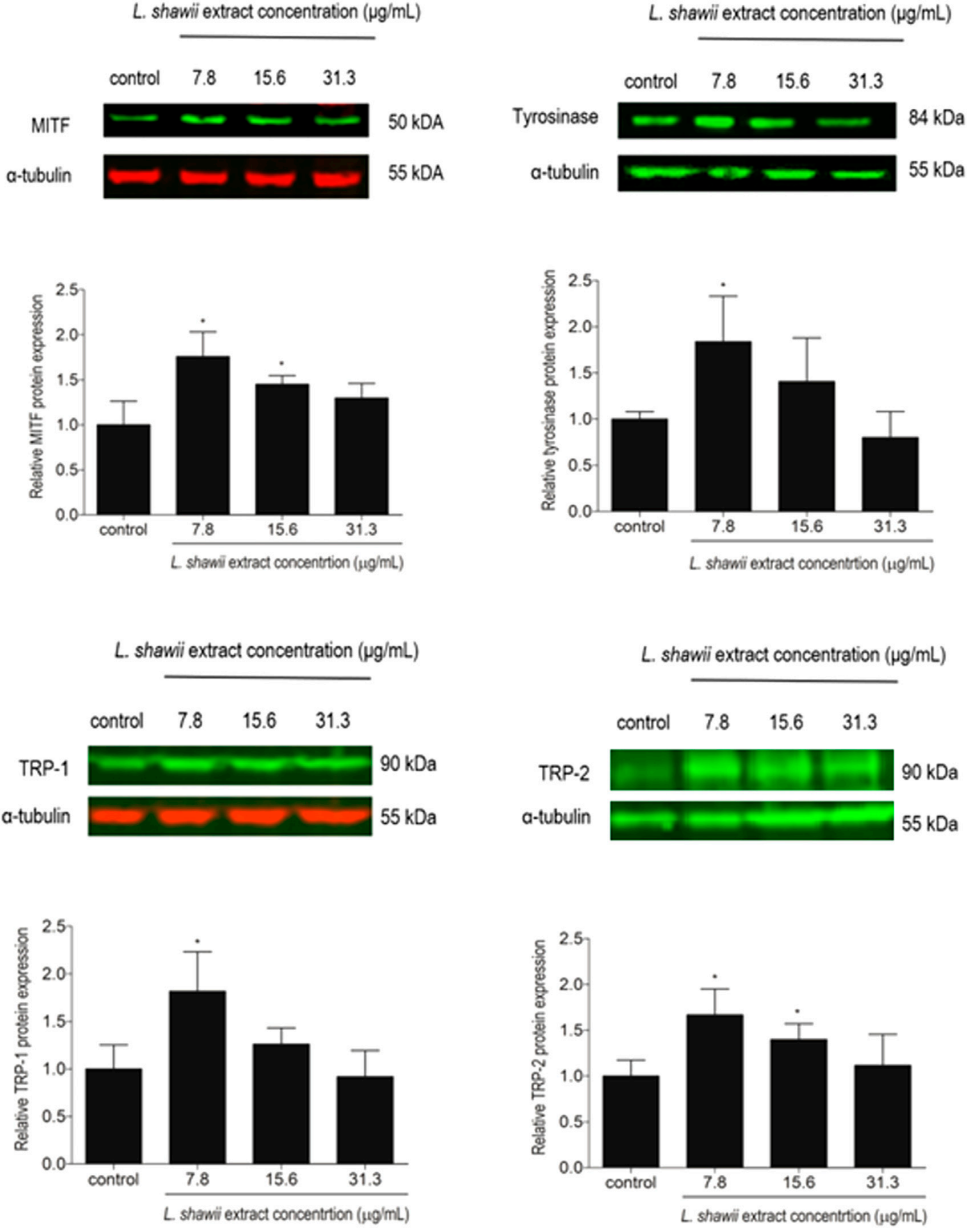
Modulatory effects of the *L. shawii* extract on the protein expression levels of MITF, tyrosinase, TRP-1, and TRP-2 melanogenesis-related proteins using Western blot analysis. Representative Western blots showing the variation of melanogenesis-related protein expression levels in melanocytes after exposure to various concentrations (7.8–31.3 μg/mL) of the *L. shawii* extract. The bar graphs show the relative expression of each melanogenesis-related protein calculated as the ratio to the housekeeping protein α-tubulin, compared to the untreated cells. The results are presented as mean ± SD of three independent experiments. *p < 0.05 signify a statistically significant difference compared with the control.

The expression of melanogenesis-related proteins MITF, tyrosinase, TRP-1, and TRP-2, well-known as markers for melanosome development and maturation contributing to melanin biosynthesis, was assessed by Western blot analysis. After 48 h of melanocyte exposure to various concentrations of the *L. shawii* extract, a peak of stimulation of the four targeted melanogenesis-related protein expression levels was observed at the lowest tested concentration, compared to the basal protein expression level detected in the untreated melanocytes (Figure 5). A lower stimulatory effect of the extract was observed at 15.6 μg/mL on MITF and TRP-2 protein expression levels, compared to the basal protein expression level detected in the untreated melanocytes (Figure 5). At the highest tested concentration of the methanolic extract, no change in the targeted protein expression levels was detected in the treated melanocytes compared to the basal protein expression level detected in the untreated melanocytes (Figure 5).

### 3.4 Identification of *L. shawii* extract-derived metabolites

**FIGURE 6:**
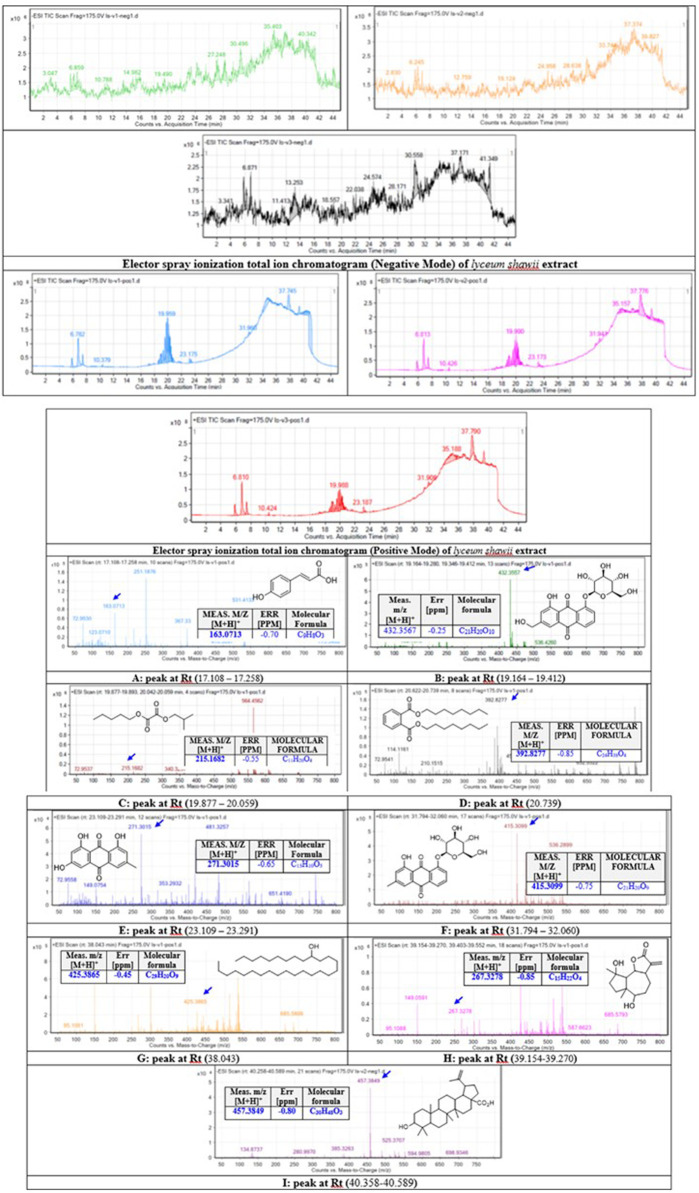
Base peak chromatograms of the *L. shawii* methanolic extract were extracted, and tentatively identified secondary metabolites are as follows: P-coumaric acid (4-hydroxycinnamic acid) (Pei et al., 2016) **(A),** aloe-emodin (Rehman et al., 2018) **(B),** oxalic acid isobutyl pentyl ester (2-methylpropyl pentyl ethanedioate) (Singariya et al., 2015) **(C),** di-n-octyl phthalate (dioctyl 1,2 benzenedicarboxylate) (DeAngelo et al., 1986) **(D)**, apigenin (4,5,6-trihydroxy flavone) (Shukla and Gupta, 2010) **(E)**, chrysophanol-8-O-β-D-glucoside (Rehman et al., 2016) **(F)**, nonacosan-10-ol (Yao et al., 2011) **(G)**, lyciumate (Rehman et al., 2019) **(H)**, betulinic acid (Yao et al., 2011) **(I)**, and *m/z* implies measured *m/z*.

The methanolic extract of *L. shawii* was subjected to total ion current (TIC) spectra raw data to reveal its derivative metabolites (Figure 6). The data-analysis program MassHunter (Agilent Technologies) was used for qualitative and quantitative analysis. After conducting a mass screening on the following spectra (Figure 6), the main chemical features were extracted from the LC-QTOF data using the molecular feature extraction (MFE) algorithm and the recursive analysis workflow. The molecular features were extracted by screening the detected nodes at various retention times per minute, with a minimum intensity of 6,000 counts, and aligned with previously detected compounds considering adducts ([M + H]^+^, [M+2H]^+^, and [M-H]^-^). The identified derivative metabolites were (peak A) P-coumaric acid (4-hydroxycinnamic acid) (Pei et al., 2016); (peak B) aloe-emodin (Rehman et al., 2018); (peak C) oxalic acid isobutyl pentyl ester (2-methylpropyl pentyl ethanedioate) (Singariya et al., 2015); (peak D) di-n-octyl phthalate (dioctyl 1,2 benzenedicarboxylate) (DeAngelo et al., 1986); (peak E) apigenin (4,5,6-trihydroxy flavone) (Shukla and Gupta, 2010); (peak F) chrysophanol-8-O-β-D-glucoside (Rehman et al., 2016); (peak G) nonacosan-10-ol, (Yao et al., 2011); (peak H) lyciumate (Rehman et al., 2019); and (peak I) betulinic acid (Yao et al., 2011), and means *m/z* implies measured *m/z.*

### 3.5 Computational docking study

**FIGURE 7:**
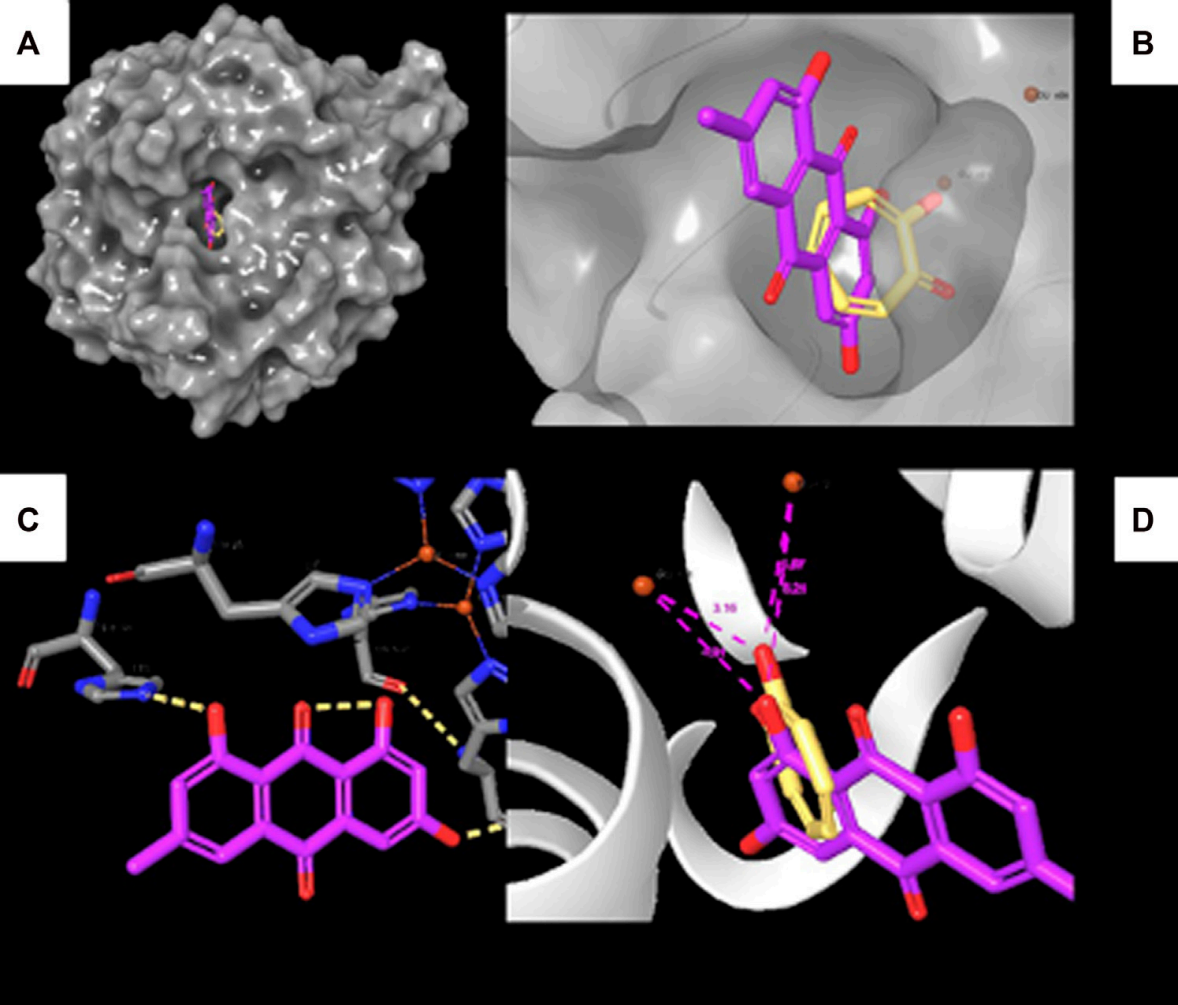
Molecular docking interactions of Metabolite 5 with the tyrosinase crystal structure. **(A)** Surface representation of tyrosinase with Metabolite 5 (violet) and tropolone (yellow) at the active site. **(B)** Active site of tyrosinase with Metabolite 5 (violet) and tropolone (yellow) with two copper ions. **(C)** Molecular interactions between Metabolite 5 and amino acid residues (dotted yellow lines represent hydrogen bonds). **(D)** Measured distance between two copper ions and oxygen atoms in Metabolite 5 and tropolone chemical structure.

To reveal the binding mode of the metabolites with tyrosinase, the Maestro Schrödinger software was used. The docking scores for all nine metabolites with their amino acid interactions are summarized in Table 2. The docking scores range from −5.601 to −0.620, with some metabolites having no docking scores (e.g., Metabolites 6, 7, and 9) (Table 2). The tyrosinase structure contains two copper ions, each connected to three histidine (HIS) residues, which are mainly responsible for the hydroxylation of monophenols, consequently leading to melanin production (Yoon et al., 2009; Zeng et al., 2020). The direct chelation into the copper ions could result in decreased and suppressed enzyme activity (Olivares et al., 2009; Zeng et al., 2020). As summarized in Table 2 and depicted in Figures 7A–C, Metabolite 5 exhibited the highest docking score (−5.601 kcal/mol) and occupied the binding pocket of tropolone by forming two hydrogen bond interactions with the methionine residue (MET) 280 and HIS 244 only. It is worth mentioning that Metabolite 5 showed no interaction with the copper ions, and the oxygen atom displayed a greater distance to the copper ions relative to tropolone (Figure 7D). Metabolite 2 demonstrated the second-highest docking score (−4.793 kcal/mol); four hydrogen bond interactions with the amino acid residues arginine (ARG) 268, phenylalanine (PHE) 264, glutamic acid (GLU) 256, and histidine (HIS) 259 and chelation to the two copper ions were present in the active sites CU 400 and CU 401.

**TABLE 2:** Glide docking scores and amino acid interactions of identified metabolites with the tyrosinase crystal structure.

Metabolite number	Glide docking score (kcal/mol)	Interactions with amino acid residues
1	−3.855	HIS 85, HIS 244, and CU 401
2	−4.793	ARG 268, PHE 264, GLU 256, HIS 259, CU 400, and CU 401
3	−0.620	-
4	−2.920	PHE 264
5	−5.601	MET 280 and HIS 244
6	-	-
7	-	-
8	−4.503	ASN 260
9	-	-

### 3.6 Predictions of the ADME properties for the metabolite-derived *L. shawii* methanolic extract

**FIGURE 8:**
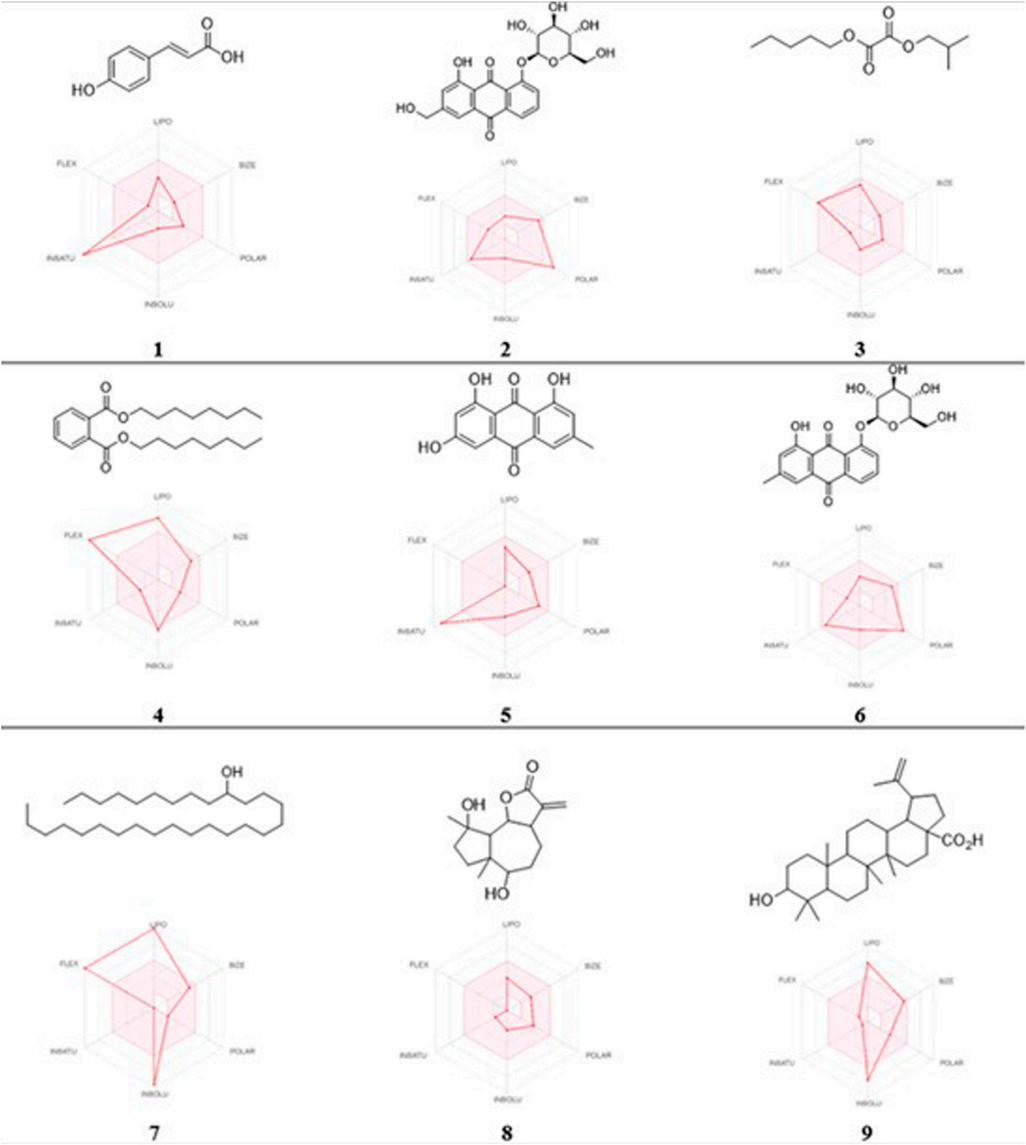
2D chemical structures and radar pharmacokinetic representation for the nine identified metabolites from the *L. shawii* methanolic extract using SwissADME Webserver. LIPO: lipophilicity, size: molecular weight, POLAR: solubility, INSOLU: insolubility, INSATU: insaturation, and FLEX: flexibility. The parameters within the colored zone are favored for orally active drugs.

In this study, the nine metabolites identified from the *L. shawii* methanolic extract were evaluated for their ADME properties using the SwissADME webserver and the QikProp tool, as summarized in Table 3 and Figure 8. All nine screened metabolites demonstrated a molecular weight of less than 500 g/mol. Of the metabolites, Metabolites 4, 7, and 9 exhibited a high log *p*-value, indicating high lipophilicity and low water solubility, as these two properties are inter-correlated. Only three (Metabolites 1, 3, and 8) of the nine metabolites were predicted to cross the BBB; thus, potential central nervous system (CNS) side effects could occur. Last, four metabolites (Metabolites 2, 6, 7, and 9) showed low oral absorption due to the violation of Lipinski’s rule of 5 (ROF) for oral drug bioavailability. Of note, Metabolite 5 demonstrated an excellent ADME profile in which all properties were within the recommended range according to ROF.

**TABLE 3:** Predicted pharmacokinetic parameters for identified metabolites derived from *Lycium shawii L.* methanolic extract using the SwissADME and QikProp tools.

Metabolite number	Molecular weight (g/mol)		Log po/w (lipophilicity)		Log S (solubility)		BBB permeant (CNS effects)		GI absorption (oral bioavailability)		Rule of five (ROF)
	SwissADME	QikProp	SwissADME	QikProp	SwissADME	QikProp	SwissADME	QikProp	SwissADME	QikProp	SwissADME
1	164.16	164.16	1.38	1.415	−1.28	−1.63	Yes	−2	High	67.30	Yes; 0 violation
					Soluble						
2	432.38	432.38	−1.31	−1.418	−2.21	−2.47	No	−2	Low	20.83	Yes; 1 violation: NH or OH>5
					Soluble						
3	216.27	216.27	1.92	2.363	−2.50	−3.56	Yes	−1	High	93.80	Yes; 0 violation
					Soluble						
4	390.56	390.56	6.72	6.248	−8.15	−5.87	No	−2	High	100	Yes; 1 violation: MLOGP>4.15
					Poorly soluble						
5	270.24	270.24	1.89	1.234	−3.91	−3.03	No	−2	High	68.02	Yes; 0 violation
					Soluble						
6	416.38	416.38	−0.35	−0.498	−2.79	−2.93	No	−2	Low	47.87	Yes; 0 violation
					Soluble						
7	424.79	424.79	10.53	10.392	−10.55	−11.35	No	−2	Low	100	Yes; 1 violation: MLOGP>4.15
					Insoluble						
8	266.33	266.33	1.41	1.503	−1.90	−2.67	Yes	0	High	85.91	Yes; 0 violation
					Soluble						
9	456.70	456.70	7.09	6.202	−5.70	−6.91	No	−1	Low	93.13	Yes; 1 violation: MLOGP>4.15
					Moderately soluble						

### 3.7 Cytochrome P450 (CYP) enzyme inhibition profiling

Using the SwissADME tool, we determined the possible drug–herbal interactions by computing the effects of the nine metabolites on multiple CYP enzymes, including CYP1A2, CYP2C19, CYP2C9, CYP2D6, and CYP3A4. Of the nine metabolites, only Metabolites 5 and 9 were predicted to inhibit CYP1A2, CYP3A4, and CYP2C9 (Table 4). The *L. shawii* methanolic extract had minimal effects on drug metabolism.

**TABLE 4:** Predicted effects of the metabolite-derived *L. shawii* extract on CYP enzyme inhibition using the SwissADME webserver.

Metabolite number	CYP1A2	CYP2C19	CYP2C9	CYP2D6	CYP3A4
1	No	No	No	No	No
2	No	No	No	No	No
3	No	No	No	No	No
4	No	No	No	No	No
5	Yes	No	No	No	Yes
6	No	No	No	No	No
7	No	No	No	No	No
8	No	No	No	No	No
9	No	No	Yes	No	No

### 3.8 Organ toxicity predictions

Multiple toxicity endpoints were evaluated for the nine metabolites, including hepatotoxicity, carcinogenicity, immunotoxicity, mutagenicity, and cytotoxicity, using the ProTox-II web server. Interestingly, all the metabolites were inactive as hepatotoxic and cytotoxic, four of the nine were carcinogens (Metabolites 1, 3, 4, and 9), four were immunotoxic (Metabolites 2, 6, 8, and 9), and three were computed to be active as mutagenic (Metabolites 2, 5, and 6), as summarized in Table 5.

**TABLE 5:** Organ and endpoint toxicity predictions using the ProTox-II web server.

Metabolite number	Classification				
	Organ toxicity (% probability)	Toxicity endpoint (% probability)			
	Hepatotoxicity	Carcinogenicity	Immunotoxicity	Mutagenicity	Cytotoxicity
1	Inactive (0.51)	Active (0.50)	Inactive (0.91)	Inactive (0.93)	Inactive (0.81)
2	Inactive (0.87)	Inactive (0.80)	Active (0.92)	Active (0.87)	Inactive (0.75)
3	Inactive (0.85)	Active (0.72)	Inactive (0.95)	Inactive (0.71)	Inactive (0.85)
4	Inactive (0.84)	Active (0.75)	Inactive (0.69)	Inactive (0.99)	Inactive (0.89)
5	Inactive (0.70)	Inactive (0.79)	Inactive (0.69)	Active (0.93)	Inactive (0.96)
6	Inactive (0.85)	Inactive (0.84)	Active (0.98)	Active (0.91)	Inactive (0.77)
7	Inactive (0.70)	Inactive (0.68)	Inactive (0.95)	Inactive (0.98)	Inactive (0.78)
8	Inactive (0.76)	Inactive (0.66)	Active (0.99)	Inactive (0.63)	Inactive (0.71)
9	Inactive (0.54)	Active (0.53)	Active (0.74)	Inactive (0.71)	Inactive (0.97)

## 4 Discussion

The search for novel therapeutic strategies for melanin production-based pigmentation of skin diseases, such as vitiligo, is still in demand (Narayan et al., 2021). To date, there is no first-line therapy for vitiligo. However, a few therapies are used, including ultraviolet (UV) light, cosmetic coverage, topical corticosteroid creams, and surgery for the transplantation of autologous melanocytes (Abdel-Malek et al., 2020). Emerging drugs such as calcineurin inhibitors, topical and oral Janus kinase (JAK) inhibitors, topical immunosuppressants, macrolide immunomodulators (e.g., tacrolimus and pimecrolimus), and phytochemicals have been promising in conjunction with NB-UVB (Chen et al., 2016; Konstantinova et al., 2019; Karagaiah et al., 2020). Natural products, including plant-based natural products, serve as a source of new therapies for numerous illnesses, including skin disorders such as vitiligo. In this study, after screening various methanolic extracts derived from Saudi Arabian plants, based on their impact on melanocyte proliferation, the *L. shawii* methanolic extract efficiently stimulated melanocyte functions, including migration and melanogenesis. Several cell- and molecular-based investigations were performed with the *L. shawii* extract, revealing its stimulatory effects on melanocyte regeneration, characterized by an increase in melanosome maturation, melanin production, and the upregulation of melanogenesis-related protein expression levels. Identification of the metabolite-derived *L. shawii* extract and the molecular docking study revealed the molecular interactions between identified Metabolite 5 and the tyrosinase active site, predicting an increase in tyrosinase activity, and subsequently melanogenesis. These findings suggest the *L. shawii* methanolic extract as a potential alternative therapy for vitiligo.

After the collection of human foreskin tissues, their culture, and the characterization of the primary human melanocytes, a screening of the methanolic plant extracts was conducted based on their stimulatory effect on melanocyte proliferation, a crucial cell event associated with melanin production. The cell growth was evaluated with the MTT assay. Three methanolic extracts prepared from the *A. fragrantissima* aerial part, *C. comosum* leaves, and *R. stricta* leaves showed cytotoxicity. No change in cell growth was observed after the cell exposure to a methanolic extract of the *A. hierochuntica* aerial part. An increase in cell growth was observed at the high concentration (i.e., 125.0 µg/mL) of the methanolic extract of the *C. procera* aerial part. The methanolic extract of *L. shawii* leaves at the lowest tested concentration (i.e., 7.8 μg/mL), displayed a significant increase in human melanocyte proliferation without causing cytotoxicity at high concentrations (i.e., 500.0 μg/mL), and its effect was evaluated using additional experiments related to melanogenesis. Of note, skin melanocyte functions such as migration, proliferation, and melanogenesis are regulated by a complex network of extrinsic and intrinsic signaling pathways, including the mitogen-activated protein kinase (MAPK) pathway and its downstream effector MITF, a transcription factor of melanogenesis-related gene expression (Zhou et al., 2022). In addition, *L. shawii*, also known as the Arabian boxthorn, is a thorny shrub mainly found in the Arabian Peninsula. The *L. shawii*’s leaves and edible berries are used to treat numerous ailments, including diabetes, hypertension, parasitic diseases, mouth sores, coughs, and backache, and are used as a laxative (Dahech et al., 2013). A comprehensive study of Saudi Arabian herbal medicine reported the common use of plant extracts for melanocyte function stimulation, mostly members of the *Amaranthaceae* and *Euphorbiaceae* families, with beneficial effects in topical applications for skin disease treatment (Almoshari, 2022). In agreement with our study, plant leaf extracts have the strongest pigment-stimulatory effects, compared to other plant parts (Almoshari, 2022).

The melanogenesis process for melanin production occurs in the ultra-structures of lysosome-like subcellular melanosomes, in which the biochemical reactions driven by tyrosinase, TRP-1, and TRP-2 result in the hydroxylation of L-tyrosine to L-DOPA and in catalyzing eumelanin-producing reactions (D’Mello et al., 2016; Tian et al., 2021). At the structural level, melanin-producing melanosomes are classified at the different stages of maturation based on melanin production (Tian et al., 2021). Melanogenic enzymes, including tyrosinase and their major targets TRP-1 and TRP-2, are synthesized by the transcription factor MITF (D’Mello et al., 2016; Tian et al., 2021). Stimulation of melanogenesis is accompanied by an increase in the number of melanosomes, which is subsequently correlated with increased melanin production, associated with the upregulation of MITF and melanogenic enzymes tyrosinase, TRP-1, and TRP-2 (Tian et al., 2021). In this study, the primary culture of human melanocytes exposed to increasing concentrations (i.e., 7.8–31.3 μg/mL) of a methanolic extract of *L. shawii* leaves increased the number of melanosomes at later stages of maturation, which reflected and confirmed the increase in melanin production, observed in the melanocytes exposed to the highest concentration of the methanolic extract. However, it was noteworthy that the *L. shawii* methanolic extract upregulated MITF, tyrosinase, TRP-1, and TRP-2 protein expression at the lowest concentration and then slowly decreased the protein expression levels to the basal level detected in the untreated melanocytes. The gradual decrease in MITF, tyrosinase, TRP-1, and TRP-2 expression levels detected in melanocytes exposed to higher concentrations of methanolic extracts could be explained by protein degradation in the melanosomes, which contain Rab small GTPases such as Rab7B/42 and endolysosomal membranes (Marubashi and Fukuda, 2020; Netcharoensirisuk et al., 2021). Thus, an assessment of the cellular activity of Rab7B/42 expressed in the melanocytes, exposed to various concentrations of the *L. shawii* methanolic extract, would be of interest.

To examine *L. shawii* extract-derived bioactive metabolites as potential drugs for vitiligo treatment, a chemical analysis was performed, followed by a molecular docking analysis for studying the molecular interaction between the identified metabolites and tyrosinase, the key enzyme leading to melanin production. Screening the base peak chromatogram of the *L. shawii* methanolic extract tentatively identified the following secondary metabolites: (peak A) P-coumaric acid (4-hydroxycinnamic acid); (peak B) aloe-emodin; (peak C) oxalic acid isobutyl pentyl ester (2-methylpropyl pentyl ethanedioate); (peak D) di-n-octyl phthalate (dioctyl 1,2 benzenedicarboxylate); (peak E) apigenin (4,5,6-trihydroxy flavone); (peak F) chrysophanol-8-O-β-D-glucoside; (peak G) nonacosan-10-ol; (peak H) lyciumate; and (peak I) betulinic acid. Molecular docking is an efficient, rapid, and widely used approach for assessing and evaluating the protein–ligand interactions (Kitchen et al., 2004). In this study, the nine identified metabolites of the *L. shawii* methanolic extract were docked into the tyrosinase crystal structure to investigate the probable molecular interactions. Metabolite 5, identified as apigenin (4,5,6-trihydroxyflavone), exhibited the highest docking score, followed by Metabolite 2. The docking analyses revealed that Metabolite 5 formed only two hydrogen bond interactions with active-site residues and no copper chelation, confirming that there is no inhibition of the tyrosinase enzyme activity. These findings predicted an increase in the tyrosinase enzyme activity elicited by the *L. shawii* extract, which was similar to a study that demonstrated that emodin increases tyrosinase activity and melanin production *in vitro* (Kim and Kim, 2022). The docking results provide additional clues that Metabolite 5 is responsible for enhancing melanin synthesis and production *via* the increased activity of tyrosinase, the melanogenesis activator (Guan et al., 2008; Kim and Kim, 2022).

The ADME prediction study revealed that most of the metabolites possess a desirable pharmaceutical profile and are considered drug-like compounds, in particular, Metabolite 5, which was within the recommended range of the pharmacokinetic description required for orally bioavailable drugs, according to Lipinski’s ROF (Lipinski, 2000; Lipinski, 2004). The majority of the identified metabolites were inactive as CYP enzyme inhibitors, except for a few that pose a potential risk for adverse effects and/or possible drug interactions (Huang et al., 2008). Most of the metabolites were predicted to possess potential undesirable toxicity that could occur with the consumption of high doses and long-term use (Dong et al., 2016), except for Metabolite 7, which was inactive in all the assessed toxicity endpoints.

## 5 Conclusion

Our findings described the promising pro-melanogenic activities of the *L. shawii* methanolic extract on primary cultures of human melanocytes. The pro-melanogenic effects were revealed by the increase in melanocyte proliferation and migration, enhancement of melanosome formation and maturation, and upregulation of melanogenesis-related proteins, including MITF, tyrosinase, TRP-1, and TRP-2. After the chemical analysis and metabolite identification of the *L. shawii* methanolic extract, a computational docking approach revealed that the identified Metabolite 5 (i.e., apigenin) exhibited the highest docking score with the tyrosinase active site, predicting an increase in melanogenic activity, and subsequently, melanin formation. This prediction remains to be confirmed using biochemical assays (i.e., tyrosinase activity) and *in vivo* studies (i.e., pro-melanogenic activities).

## Figures and Tables

**Table:** 

**2D**	Two-dimensional
**3D**	Three-dimensional
**BBB**	Blood–brain barrier
**CCD**	Charge-coupled device
**CNS**	Central nervous system
**CO**_**2**_	Carbon dioxide
**CYP**	Cytochrome P450
**DMEM**	Dulbecco's modified Eagle medium
**DMSO**	Dimethyl sulfoxide
**DOPA**	Dihydroxyphenylalanine
**EDTA**	Ethylenediaminetetraacetic acid
**FBS**	Fetal bovine serum
**HIS**	Histidine
**HPLC**	High-performance liquid chromatography
**H**_**2**_ **O**_**2**_	Hydrogen peroxide
**IC50**	Half-maximal inhibitory concentration
**MFE**	Molecular feature extraction
**MITF**	Microphthalmia-associated transcription factor
**MTT**	3-[4,5-dimethylthiazol-2-yl]-2,5-diphenyltetrazolium bromide
**NaOH**	Sodium hydroxide
**NB-UVB**	Narrowband ultraviolet B
**PBS**	Phosphate-buffered saline
**PDB**	Protein Data Bank
**PUVA**	Psoralen ultraviolet A
**QTOF**	Quadrupole time-of-flight
**ROF**	Rule of five
**RT-qPCR**	Reverse transcription-quantitative polymerase chain reaction
**SD**	Standard deviation
**SMILES**	Simplified molecular-input line-entry system
**TEM**	Transmission electron microscopy
**TIC**	Total ion current
**TRP**	Tyrosinase-related protein
**TYR**	Tyrosinase
**UV**	Ultraviolet

## Data Availability

The raw data supporting the conclusion of this article will be made available by the authors, without undue reservation.
